# Essential oil blend supplementation in the milk replacer of dairy calves: Performance and health

**DOI:** 10.1371/journal.pone.0291038

**Published:** 2023-10-03

**Authors:** Marina G. Coelho, Ana Paula da Silva, Ariany F. de Toledo, Amanda M. Cezar, Cristiane R. Tomaluski, Rayane D. F. Barboza, Gercino F. Virginio Júnior, Ricardo P. Manzano, Carla M. M. Bittar

**Affiliations:** 1 Department of Animal Science, Luiz de Queiroz College of Agriculture, University of São Paulo, Piracicaba, São Paulo, Brazil; 2 Minas Gerais Agricultural Research Agency, Experimental Field of Montes Claros, Montes Claros, Minas Gerais, Brazil; 3 Nutripec Consulting, São Paulo, Brazil; University of Illinois, UNITED STATES

## Abstract

Supplementation of dairy calves with essential oils in the liquid diet can reduce the incidence and severity of infectious diseases and promote better performance. Our objective was to evaluate an essential oil blend containing peppermint, eucalyptus, and menthol crystals and its effect on performance and health during the pre and postweaning period of dairy calves. At birth, 40 dairy calves (34 males and 6 females) were blocked according to sex and birth weight, randomly assigned to one of two treatments–commercial milk replacer diluted at 14% (MR) and the same liquid diet plus essential oil blend (MREO) at a dose of 4 mL per calf per day, divided into two meals during the first 28 days of life. Calves were individually housed and fed 6 L/d of the liquid diet, divided into two meals, and received starter concentrate and water ad libitum. Weaning was gradually reduced by 1L per day at d 51 until complete weaning at 56 days. After weaning, calves were evaluated until 70 days of age, when the experimental period ended. Intake, fecal, and health scores were evaluated daily, weight and body measures were recorded weekly, and blood parameters were evaluated at weeks 1, 2, 3, 4, and 8. Calves fed MREO tended to have higher total dry matter intake during the preweaning period. Furthermore, MREO calves had lower health scores and fewer days with a health score ≥ 4 (suggestive of bronchopneumonia), tended to lower coughing days and fewer veterinary interventions preweaning, and tended to lower health scores postweaning. The supplementation with a blend of essential oils containing peppermint, eucalyptus, and menthol crystals can reduce respiratory problems. Further studies are needed to investigate the dose and the supplementation period.

## Introduction

An appropriate calf-rearing program should consider nutritional requirements, stress reduction, and health management to optimize the calf’s health status and overall performance. Furthermore, the indicators of this period relate not only to the success of the calf’s feeding program but also to its subsequent milk production [1, 2].

Nutritional problems and neonatal diseases, especially diarrhea and respiratory syndrome, are among the examples that cause negative impacts on the productive life of replacement females. These problems can act as stress factors, decreasing immunity, increasing susceptibility to other disorders, and increasing mortality rates [3, 4].

Therefore, effective strategies that support proper nutrition and improve both development and health are essential to reduce morbidity and mortality and accelerate calf development [5]. The most common approach for this purpose has been antibiotic growth promoters; however, their use has been severely criticized and banned in many countries [6]. As an alternative to antibiotics, feed additives can modulate the ruminal and intestinal microbiome, modify ruminal fermentation, decrease the incidence of diarrhea, modulate the immune response, and consequently improve gut health and performance [7].

The importance of some plant-based feed additives (e.g., phytobiotics, plant extracts, and essential oils) becomes evident since the secondary plant compounds have among their properties antiseptic and antimicrobial activities [8, 9]. These activities interfere with the functioning of bacterial, fungal, and protozoan cells [9], with similar efficiency as antibiotics to treat some diseases. The compounds also prevent oxidative stress [10], can alter the phagocytic activity of leukocytes, and inhibit the complement system [8].

Blends of essential oils (BEO) in dairy calf feed have been evaluated in several studies, either by different combinations of essential oils [11–15], different dosages [12, 16, 17] or by different routes of ingestion (e.g., direct ingestion, liquid diet or starter) [13, 15, 18]. Literature reports beneficial effects on performance [12–14, 17, 18], ruminal fermentation [11, 15, 16], modulation of the ruminal microbiome [16], lower fecal score [13, 15, 17, 18], and higher concentration of immunoglobulins [17].

Although all beneficial effects have been reported, none of the studies have described the effect of the different BEOs on respiratory diseases in dairy calves. We hypothesize that using BEO, containing eucalyptus oil, peppermint oil, and menthol crystals, may benefit the dairy calves’ health and, consequently, their performance. This study aimed to evaluate whether supplementation of a commercial blend of essential oils in milk replacer (MREO) affects the performance and health of dairy calves in pre- and postweaning periods.

## Material and methods

The Ethics Committee for Research on Animals of the "Luiz de Queiroz" College of Agriculture / the University of São Paulo (ESALQ/USP) approved all procedures involving all animals in this study (Protocol No. 9808150621).

### Animals, experimental design and treatments

The study was conducted on the Experimental Calf Unit "Evilásio de Camargo", from the Animal Science Department of ESALQ/USP, located at Piracicaba–São Paulo, from September 2021 to March 2022. Forty newborn Holstein male (n = 34; 32.84 ± 1.54 Kg) and female (n = 6; 28.22 ± 0.81 Kg) calves were used in a randomized block design. Calves were blocked according to sex, date of birth, and birth weight. The calves were fed two doses of colostrum replacer (SCCL®, Saskatoon, Canada), providing 200 g IgG per calf in the first 6 h of life [19]. The serum Brix values averaged 9.2% Brix ± 1.16 (SEM), considered adequate for the immunity transfer according to the recommendations of Godden et al. [20].

All calves received 6 L/d of commercial milk replacer (Nurture Prime, Cargill Alimentos Ltda., Itapira, SP, Brazil) diluted at 14%, offered in a teat-bucket, divided into two meals (0700 and 1700). The milk replacer was diluted in tap water at 41–42°C, which after mixing, reached a temperature of 38–39°C for feeding.

A blend of essential oils (BronchoVest, Biochem, Germany) was added or not to the milk replacer, thus constituting one of two treatments: 1) Milk replacer without essential oil (MR; n = 20); and 2) Milk replacer plus 2 mL essential oil/feeding (MREO; n = 20); hence the total daily dose was 4 mL during the first 28 days of life. From day 29 to day 56, the animals in the MREO treatment received milk replacer but no added BEO.

The BEO comprises three sources of the two active principles, cineol and menthol: eucalyptus oil (157.9 g/L), peppermint oil (32 g/L), and menthol crystals (55 g/L). The BEO was diluted in distilled water to a 1:10 concentration before use, as recommended by the manufacturer (Biochem, Germany), stored in an amber glass bottle, and kept in a dark place in room temperature until feeding. The diluted BEO (2 mL) was added to the milk replacer (3 L) using a plastic pipette and immediately offered to the calf of the MREO treatment. So, calves were supplemented with 4 mL of the BEO every day until 28 d of age. The supplementation period was chosen because this is the period of occurrence for most health problems.

All calves were weaned gradually starting on day 51, reducing 1 L per day until complete weaning at 56 days, the so-called preweaning phase. After weaning, calves continued to be evaluated until 70 days of age, when the experimental period ended.

### Calf housing and intake

Immediately after birth, all calves were housed in individual suspended cages (113 x 140 cm) with sawdust beds in a ventilated barn during the first 21 days of life. From day 22 of age, calves were moved to individual external wood hutches (1.35 m high, 1 m wide, and 1.4 m deep) distributed on trimmed grass and contained by collars and chains (2 m).

Calves had free access to the commercial starter (Startmilk 20, Nutrimax Nutrição Animal Ltda., Salto de Pirapora, SP, Brazil) until reaching a maximum intake of 2.5 kg per day. The starter was provided immediately after the liquid diet was fed in the morning. The daily intake was calculated by the difference between the offered and orts. After weaning, the calves received coast-cross hay free-choice. The total dry matter intake (TDMI, milk replacer plus starter at preweaning, and starter plus hay, at postweaning) were determined daily. Average daily gain (ADG) and feed efficiency (FE; kg of BW gain/kg of TDMI) were calculated for preweaning (1–56 d) and postweaning (57–70 d) periods.

### Sampling and laboratory analysis

Samples of starter, hay, and milk replacer were collected monthly to determine the chemical composition of the offered diet. The samples were ground in a 1 mm Wiley sieve mill (Marconi, Piracicaba, Brazil). The dry matter (DM) was determined by drying samples in an oven at 105°C for 24 h (AOAC method 925.40) and ash by incinerating samples in a muffle furnace at 550°C for 4 h (AOAC method 942.05). Neutral detergent fiber (NDF) analysis was performed using an Ankom 2000 fiber analyzer (Ankom Tech. Corp.; Fairport, NY), heat stable α-amylase and sodium sulfite were included in NDF analysis, residues were incinerated to calculate ash-free NDF [21]. Total nitrogen concentration was determined using the Leco TruMac N apparatus (Leco Corporation, St. Joseph, MI; AOAC method 968.06), and crude protein (CP) was calculated by multiplying total nitrogen by 6.25. The ether extract (EE) concentration was determined using petroleum ether (AOAC method 920.39). The chemical composition of the diet ingredients is presented in Table 1.

**Table 1:** Chemical composition of milk replacer, starter, and hay.

	Milk replacer^1^	Calf starter^2^	Hay
Dry matter, %	89.7	88	82.5
Ash, % DM	9.7	9.9	6.2
Crude protein, % DM	22.6	21.8	9.3
Crude fat, % DM	19	3	2.5
aNDF^3^, % DM	-	28.4	74.7

^1^Nurture Prime, Cargill Alimentos Ltda., Itapira, SP, Brazil

^2^Start milk 20% Nutrimax Nutrição Animal Ltda., Salto de Pirapora, SP, Brazil

^3^aNDF, ash free Neutral detergent fiber

### Body measurements and blood parameters

The calves were weighed at birth and weekly until the 10th week of life using a mechanical scale (ICS-300, Coimma Ltda., Dracena, SP, Brazil), always before feeding. Body measurements were performed every two weeks. Withers height and hip width were measured with a ruler with a centimeter scale (Carci, São Paulo, SP, Brazil), and the heart girth was measured with a tape with a scale in centimeters (Bovitec, São Paulo, SP, Brazil).

Blood samples (~ 10 mL) were collected at weeks 1, 2, 3, 4, and 8 of life by venipuncture of the external jugular vein 2 h after morning feeding. The samples were collected in 3 tubes: one tube containing K_3_ EDTA to evaluate the hematocrit; another tube containing sodium fluoride as an antiglycolytic and potassium EDTA as an anticoagulant to obtain plasma; and the last one with clot activator to obtain serum.

The hematocrit was analyzed by adding a blood aliquot in a capillary tube and then centrifuged in a microhematocrit at 2,000 × *g* rotation for 10 min (model SPIN 1000, Microspin, São Paulo, Brazil). Plasma and serum were obtained by centrifugation of the remaining tubes at 2,000 x g for 20 minutes at 4°C and then stored in tubes at -10°C for further analysis.

Metabolic indicators were determined using the Automatic Biochemistry System (SBA-200, CELM, Barueri, SP, Brazil). The plasma glucose and total serum protein (TSP) concentrations were analyzed using specific commercial enzyme kits (ref. 85 and ref. 99, respectively; Labtest Diagnóstica S.A., Lagoa Santa, MG, Brazil). Beta-hydroxybutyrate (BHB) was measured using the RANBUT enzyme kit (ref.: RB1007; RANDOX Laboratories—Life Sciences Ltd., Crumlin, UK).

### Fecal and health score

The fecal score was monitored daily by assessing the fluidity of feces: (1) regular and firm; (2) soft; (3) aqueous; (4) fluid, according to Larson et al. [22]. The diarrheal episode was considered when the calves had a fecal score ≥ 3 for two or more consecutive days. The diarrhea rate was calculated according to Coelho et al. [23], Diarrhea rate = (days with diarrhea ÷ days evaluated) × 100.

Calves with fecal score ≥ 3 received an oral rehydration solution (80 g dextrose, 4 g sodium bicarbonate, and 5 g sodium chloride per liter of warm water, 37°C) 4 h after the morning feeding. The rectal temperature of calves was measured daily with a digital thermometer, and fever was considered when calves had more than 39.4°C. The calves were evaluated daily using the Calf Health Scoring Criteria, developed by the University of Wisconsin-Madison, which includes the following criteria for detection of bovine respiratory disease (BRD): rectal temperature, cough, nasal secretion, ocular secretion, and head and ears position, scored from 0 to 3 according to the strength of the signals. The sum of scores ≥ 4.0 indicates positivity for BRD [4].

The calves that presented any indication of systemic involvement, such as fever, anorexia, and/or apathy, or any alteration in the pulmonary auscultation (performed weekly), received antimicrobial treatment according to the veterinary recommendation. Any other health problem was treated according to veterinary recommendation to alleviate pain or suffering.

### Statistical analysis

The experimental design was in randomized complete block design; calves were allocated in blocks according to birth weight, date of birth, and sex. Weight differences within blocks were no more than 2 kg, while the age of the calves varied to no more than 15 days. One calf from the MR treatment had to be removed from the trial because of a non-treatment-related cause. Therefore, data were analyzed considering 39 calves, 20 for the MREO and 19 for the MR treatment. Growth, intake, fecal score, and health data were analyzed as time-repeated measures using the MIXED procedure of the SAS statistical package (version 5.0, SAS Institute Inc., Cary, NC, USA) for mixed models. The model included treatment, week (age of calves), and interaction between treatment and week as fixed effects. The block effect was included in the model as a random effect and week as a repeated measure (model 1), and the subject of repeated measures used was animal (treatment).

$$Y_{ijk}=\mu +T_{i}+b_{j}+e_{ij}+S_{k}+{\left(TS\right)}_{ik}+e_{ijk}$$ (Model 1)

Where Y_ijk_ = response variable, μ = overall mean, T_i_ = fixed treatment effect, b_j_ = random block effect, e_ij_ = residual error (A), S_k_ = fixed effect of calf age (week of data collection/sample), (TS)_ik_ = fixed effect of treatment × age interaction, e_ijk_ = residual error (B). The covariance structures "composite symmetry, heterogeneous composite symmetry, autoregressive, heterogeneous autoregressive, unstructured, banded, variance components, toeplitz, antidependence, and heterogeneous toeplitz" were tested and defined according to the lowest value obtained for "Akaike’s Information Criterion corrected” (AICC).

For data pooled to create a single measure, such as diarrhea days, days with fever, days with cough, and diarrhea rate, PROC MIXED from the SAS statistical package was used according to model 2. The model included treatment as an fixed effect, and block as a random effect, and calf within treatment was included as a random effect. Means were obtained for all response variables using the LSMEANS command. F-test was used to compare treatments when there was significance in the analysis of variance.

$$Y_{ijk}=\mu +T_{i}+b_{j}+e_{ij}$$ (Model 2)

Where Y_ijk_ = response variable, μ = overall mean, T_i_ = fixed treatment effect, b_j_ = random block effect, e_ij_ = residual error. For all analyses, significance was declared at *P* ≤ 0.05 and trend when the 0.05 < *P* ≤ 0.10.

## Results

### Pre and postweaning performance

**Fig 1:**
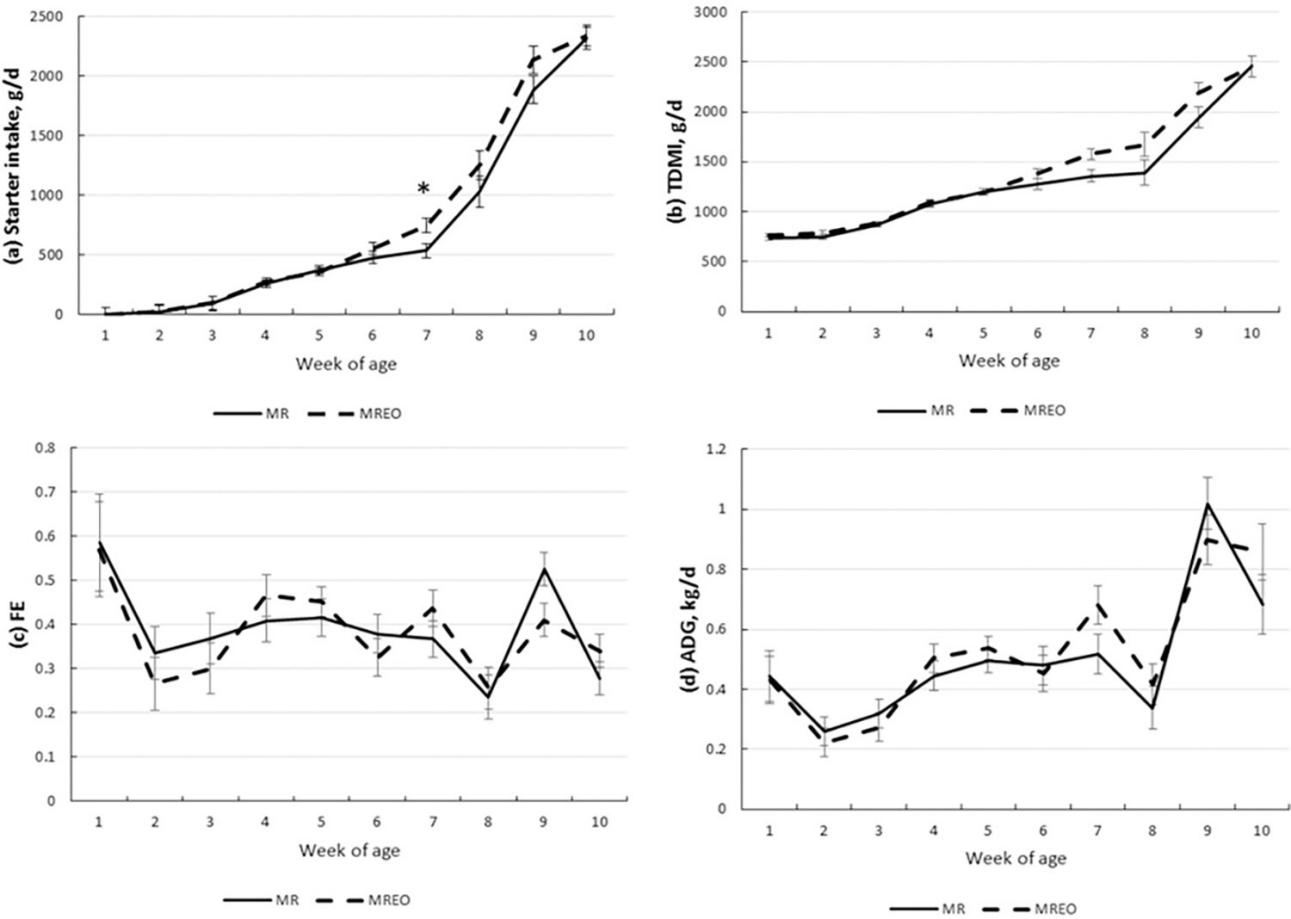
Performance according to week of age of dairy calves fed milk replacer supplemented or not with a blend of essential oils in the first 28 d of life. (a) Starter intake (g/d), (b) Total dry matter intake (g/d), (c) Feed efficiency, and (d). Average daily gain (kg/d) * denotes statistical difference P < 0.05 for the highest MREO values. MR, Milk replacer; MREO, Milk replacer with essential oil.

The intake, ADG, FE, and body measurements increased with age (*P* < 0.0001; Table 2) preweaning. There were no leftovers of liquid diet during the entire period, so all calves consumed 840 g/d, regardless of supplementation. There was a treatment by week interaction for starter intake (*P* = 0.052) with higher intake at week 7 in the MREO group (Fig 1A). A trend for higher TDMI was observed for calves in the MREO group (*P* < 0.07; Table 2). However, there was no effect of essential oil supplementation on preweaning performance.

**Table 2:** Preweaning (1–56 d of age) performance of dairy calves fed milk replacer supplemented or not with a blend of essential oils during the first 28 d of life.

	Treatment^1^		SEM^2^	*P*-value^3^		
	MR	MREO		T	A	TxA
Intake						
Starter, DM g/d	348.21	413.46	47.453	0.294	< .0001	0.052
TDMI^4^, g/d	1081.8	1170.0	33.32	0.070	< .0001	0.151
Weight, kg						
At birth	36.37	36.40	1.454	0.945	-	-
At weaning	59.06	61.54	1.834	0.280	-	-
ADG, kg/d	0.41	0.44	0.023	0.415	< .0001	0.634
Feed efficiency	0.38	0.38	0.022	0.920	< .0001	0.701
Body measurements, cm						
Withers height	82.4	82.7	0.787	0.575	< .0001	0.456
Hip width	22.6	22.6	0.315	0.927	< .0001	0.451
Heart girth	84.7	85.4	0.853	0.351	< .0001	0.277

^1^MR, Milk replacer; MREO, Milk replacer with essential oil

^2^SEM–Standard error of the mean

^3^T –Treatment effect, A—Age effect, TxA–Treatment x age interaction effect

^4^ TDMI, total dry matter intake. It includes starter and liquid diet DMI. There were no leftovers for the liquid diet offered (6 L/d or 840 g/d).

During postweaning, intake, ADG, and FE also increased with age (*P* < 0.05; Table 3). The essential oils supplementation did not affect performance (Table 3). A treatment x age interaction effect (*P* = 0.015) was observed for FE. There was a trend to larger hip width and heart girth (*P* = 0.09 and *P* = 0.10, respectively; Table 3) for calves in the MREO group.

**Table 3:** Postweaning (57–70 d of age) performance of dairy calves fed milk replacer supplemented or not with a blend of essential oils in the first 28 d of life.

	Treatment^1^		SEM^2^	*P*-value^3^		
	MR	MREO		T	A	TxA
Intake						
Starter, DM g/d	2098.8	2237.3	88.77	0.172	0.0001	0.124
Hay, DM g/d	100.1	108.1	8.10	0.453	0.0001	0.938
TDMI, g/d	2200.0	2334.5	86.94	0.186	< .0001	0.120
Weight, kg						
At weaning	59.06	61.54	1.834	0.279	-	-
Final (70 d)	70.88	74.13	2.160	0.229	-	-
ADG, kg/d	0.85	0.87	0.068	0.753	0.0270	0.084
Feed efficiency	0.39	0.37	0.028	0.406	0.0001	0.015
Body measurements, cm						
Withers height	89.5	90.5	0.847	0.322	-	-
Hip width	24.7	25.4	0.305	0.099	-	-
Heart girth	97.4	99.5	0.198	0.100	-	-

^1^MR, Milk replacer; MREO, Milk replacer with essential oil

^2^SEM–Standard error of the mean

^3^T –Treatment effect, A—Age effect, TxA–Treatment x age interaction effect

### Blood parameters

**Fig 2:**
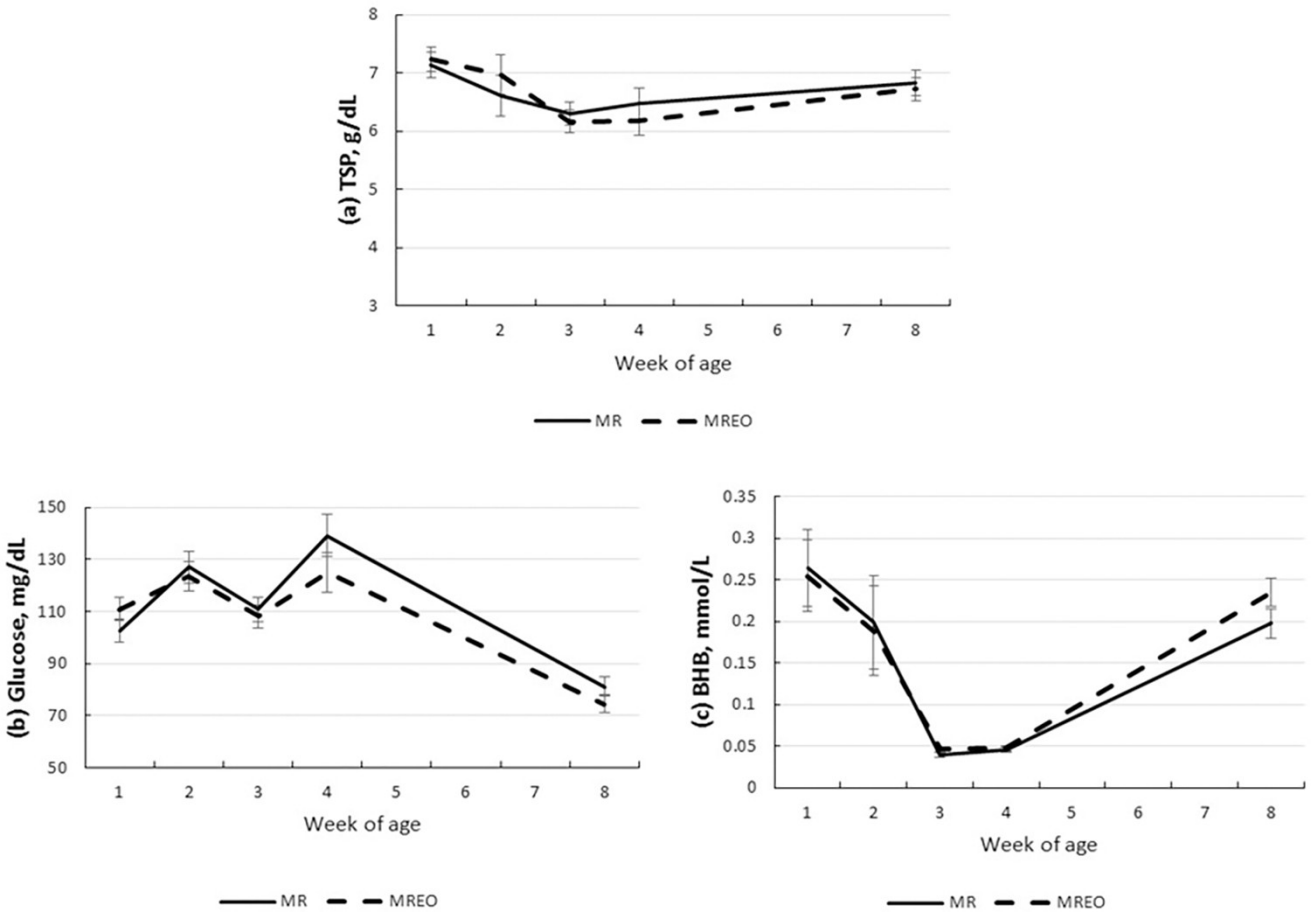
Blood parameters according to week of age of preweaning dairy calves fed milk replacer supplemented or not with a blend of essential oils in the first 28 d of life. (a) Total serum protein (g/dL), (b) Glucose (mg/dL), and (c) Beta-hydroxybutyrate (mmol/L). MR, Milk replacer; MREO, Milk replacer with essential oil.

The blood parameters evaluated did not indicate any treatment effect or treatment x age interaction (Table 4). However, all parameters were affected by age (*P* < 0.0001; Table 4); TSP decreased until week 4 (Fig 2A), while glucose decreased at week 8 (Fig 2B). Beta-hydroxybutyrate also decreased until week 3 of life, increasing at the week 8 (Fig 2C).

**Table 4:** Blood metabolites of preweaning dairy calves fed milk replacer supplemented or not with a blend of essential oils in the first 28 d of life.

	Treatment^1^		SEM^2^	*P*-value^3^		
	MR	MREO		T	A	TxA
Total serum protein, g/dL	6.67	6.66	0.144	0.935	<0.0001	0.839
Glucose, mg/dL	112.2	108.4	2.17	0.198	<0.0001	0.345
β-hydroxybutyrate, mmol/L	0.149	0.154	0.015	0.833	<0.0001	0.636

^1^MR, Milk replacer; MREO, Milk replacer with essential oil

^2^SEM–Standard error of the mean

^3^T –Treatment effect, A—Age effect, TxA–Treatment x age interaction effect

### Fecal and health score

**Fig 3:**
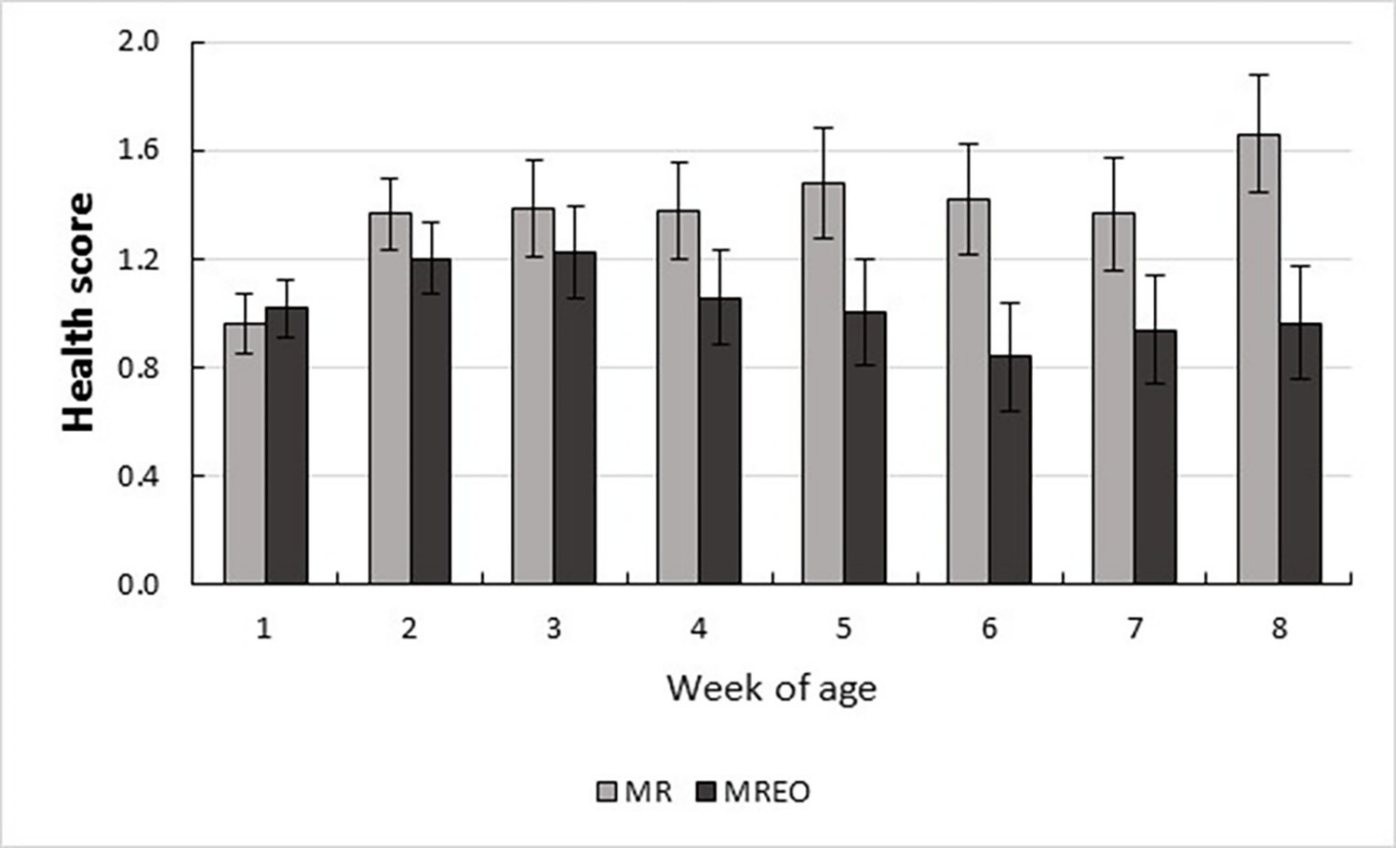
Health score according to age of preweaning (1 to 56 d) dairy calves fed milk replacer supplemented or not with a blend of essential oils in the first 28 d of life. MR, Milk replacer; MREO, Milk replacer with essential oil.

In the preweaning period, fecal score (*P* < 0.0001), health score (*P* = 0.05), and hematocrit (*P* < 0.0001) showed effects for age (Table 5). All variables evaluated showed no effect of treatment x week interaction. Nevertheless, the first three weeks of age were the period with the occurrence of most of the diarrhea, but health scores were high during all the evaluated period (Fig 3). However, health score (*P* = 0.01) and days with health score ≥ 4 (*P* = 0.05) were higher for animals without supplementation (MR; Table 5, Fig 3). Similarly, days with cough (*P* = 0.07) and the number of interventions (*P* = 0.09) tended to be higher for animals in this group. During the postweaning period, the health score (*P* = 0.07) tended to be higher for animals of the MR group.

**Table 5:** Diarrhea and health of pre (1–56 d) and postweaning (57–70 d) dairy calves fed milk replacer supplemented or not with a blend of essential oils in the first 28 d of life.

	Treatment^1^		SEM^2^	*P*-value^3^		
	MR	MROE		T	A	TxA
**Preweaning (1 to 56 d)**						
Diarrhea rate, %^4^	20.66	21.94	1.973	0.640	-	-
Fecal score	1.96	2.01	0.053	0.516	< .0001	0.138
Days with diarrhea	14.46	15.35	1.381	0.640	-	-
Days with fecal score 4	2.88	2.67	0.609	0.799	-	-
Health score^5^	1.37	1.03	0.115	0.01	0.05	0.327
Days with health score ≥ 4	4.26	1.40	1.005	0.05	-	-
Days with cough	11.52	4.80	2.532	0.07	-	-
Days with fever^6^	1.74	1.60	1.668	0.793	-	-
Interventions^7^	1.39	0.92	0.217	0.09	-	-
Hematocrit, %	25.96	26.76	0.722	0.436	< .0001	0.449
**Postweaning (57 to 70 d)**						
Health score	0.96	0.68	0.169	0.07	0.2298	0.655
Hematocrit, %	26.22	26.09	0.901	0.905	0.3922	0.512

^1^MR, Milk replacer; MREO, Milk replacer with essential oil

^2^SEM–Standard error of the mean

^3^T –Treatment effect, A—Age effect, TxA–Treatment x age interaction effect

^4^ Diarrhea rate = (days with diarrhea ÷ days evaluated) × 100

^5^ Calf Health Scoring Criteria (0 to 3) developed by the University of Wisconsin-Madison which considers rectal temperature, cough, nasal secretion, ocular secretion, and head and ears position

^6^ The calf was considered to have fever if its body temperature was > 39.4°C

^7^ Number of treatments with either antibiotics or anti-inflamatory.

## Discussion

Although no positive results were found using the BEO on the animals’ performance during the evaluated period, the health effects are significant. It is important to highlight that offering the BEO added to the milk replacer makes this study even more interesting, since the use of milk replacer in calf feeding requires attention due to nutrient levels and ingredients used in formulation. Considering the various formulations and qualities of commercial milk replacers available [24], as well as the difficulties already mentioned by Constable et al. [25] and Smith and Sissons [26], the calves MR-fed tend to have a less acidic abomasal environment, and a slower intestinal development, which directly influences their performance [27]. Indeed, these issues may have impacted the lower performance of the animals in this study compared to other studies from the same laboratory that used another type of milk replacer but with very similar nutrient levels [23, 28, 29]. According to NASEM [30] calves consuming a about 1.12 kg of DM, considering 80% from the liquid dieta and 20% from solid diet should gain around 600 g/d. Our calves were consuming a diet composed of 67% of milk replacer and 33% of concentrate, averaging 22.3% of crude protein and presented gains of 425 g/d. Nonetheless, we believe that the type of supplementation by MR is valid since it is an important liquid diet option to feed calves [31–33].

The limited supplementation period to the first 28 days of life was determined in this study due to the period of the calves’ immunological window, i.e., the period most susceptible to the main infectious diseases [4]. Also, greater economic applicability of the product was aimed. The short supplementation period may be one of the reasons why no significant changes were found from pre to postweaning performance. On the other hand, other authors supplementing calves with other BEO throughout the preweaning period also did not find significant changes in performance [11, 15].

Supplementation with essential oils is commonly performed via starter concentrate; however, intake is low in the first weeks of life [34], the period of greater occurrence of enteric diseases [4], which justifies its use via a liquid diet. There are differences in the literature regarding the acceptability of EOs, but these vary in terms of supply method, the plant used, and the extraction process [9], with further variation between species and animal category [35]. The characteristic bitter taste of these ingredients has been shown to negatively affect palatability, manifesting in food rejection [13, 36] or decreased intake [37, 38]. Soltan [39], using a similar BEO containing eucalyptus and menthol (Aeroforte, Kanters Ltd.) did not observe any difference in the liquid diet intake. In the present study, calves were supplemented through the liquid diet, and no refusals were observed during the entire preweaning period.

Additional benefits of EOs have already been reported in intake and performance [11, 14, 40, 41]. However, Soltan [39] observed that TDMI was reduced in calves supplemented with 0.09, 0.187, or 0.281 g/calf/day throughout the preweaning period with a BEO similar to the present study (eucalyptus oil, menthol, and peppermint oil). In the present study, there was a tendency to increase TDMI preweaning, which was not persistent after weaning.

Froehlich et al. [12] demonstrated that supplementing calves with a BEO (carvacrol, caryophyllene, p-cymene, cineol, terpinene, and thymol) at a dose of 2.5 g/d resulted in higher ADG and body measurements, and increased immunity compared to the control group. This increase in immunity consequently increases the health status of the animals. Despite the improved health status of the calves in our study, the performance was not affected pre or postweaning.

In the study by Salazar et al. [18] with a blend containing thymol, guaiacol, eugenol, vanilin, salicylaldehyde, and limonene, calves that received supplementation with EO during the whole preweaning period (60 d) showed no differences in performance. However, the authors observed a significant increase in ADG and FE after weaning, which shows a positive residual effect of EO up to 15 days after its interruption. In our study, MREO calves showed a tendency to improve body measurements after weaning, which may be related to the supplementation period, which was only during the first 28 days of life. In general, the calves could have presented a slightly higher ADG (500 g/d) as expected by the feeding program offering 840 g DM/d of milk replacer [30]. However, the calves in the present study were challenged by the high occurrence of tick fever and bronchopneumonia throughout the experimental period.

Some authors have established a relationship between BEO supplementation and increased immunity through increased production of immunoglobulins G and M, increasing STP in calves [41, 42]. Previous studies confirmed that IgG concentration was lower in calves with severe diarrhea than in healthy calves [43]. In our study, in addition to serum protein showing no difference between treatments, diarrhea was not a major problem in calves. Decreased glucose and increased BHBA concentrations are expected to occur with age as a response to rumen development [44].

Despite the lack of calves’ performance, there was an improvement in overall health when parameters related to the health score were considered. The score used in this study [4] is specific to the respiratory tract, and the author considers a calf that receives a score ≥ 4 positive for bronchopneumonia. The MREO calves had lower health scores, fewer days with scores ≥ 4, and tended to have fewer days with cough and fewer veterinary treatment interventions. Bampidis et al. [45] reported that oregano extract might be as effective as neomycin (an antibiotic additive) in preventing disease in calves. BEOs tend to limit the opportunity for bacterial populations to develop spontaneous resistance, making them important candidates for feed supplementation to prevent disease [46].

## Conclusions

In conclusion, although performance was not affected, calf health was influenced by supplementing the liquid diet with essential oils. The possible antimicrobial, antioxidant, and anti-inflammatory effects of these phytogenic compounds, mainly acting on the respiratory tract, result in improved health of the calves, indicated by lower health scores.

Therefore, the blend of essential oils composed of eucalyptus oil, peppermint oil, and menthol crystals may be an important alternative in preventing respiratory diseases in newborn calves. However, the dose and the supplementation period need more investigation.
